# Leaf-cutting ant fungi produce cell wall degrading pectinase complexes reminiscent of phytopathogenic fungi

**DOI:** 10.1186/1741-7007-8-156

**Published:** 2010-12-31

**Authors:** Morten Schiøtt, Adelina Rogowska-Wrzesinska, Peter Roepstorff, Jacobus J Boomsma

**Affiliations:** 1 Centre for Social Evolution, Department of Biology, University of Copenhagen, Universitetsparken 15, DK-2100 Copenhagen, Denmark; 2 Department of Biochemistry and Molecular Biology, University of Southern Denmark, Campusvej 55, DK-5230 Odense M, Denmark

## Abstract

**Background:**

Leaf-cutting (attine) ants use their own fecal material to manure fungus gardens, which consist of leaf material overgrown by hyphal threads of the basidiomycete fungus *Leucocoprinus gongylophorus *that lives in symbiosis with the ants. Previous studies have suggested that the fecal droplets contain proteins that are produced by the fungal symbiont to pass unharmed through the digestive system of the ants, so they can enhance new fungus garden growth.

**Results:**

We tested this hypothesis by using proteomics methods to determine the gene sequences of fecal proteins in *Acromyrmex echinatior *leaf-cutting ants. Seven (21%) of the 33 identified proteins were pectinolytic enzymes that originated from the fungal symbiont and which were still active in the fecal droplets produced by the ants. We show that these enzymes are found in the fecal material only when the ants had access to fungus garden food, and we used quantitative polymerase chain reaction analysis to show that the expression of six of these enzyme genes was substantially upregulated in the fungal gongylidia. These unique structures serve as food for the ants and are produced only by the evolutionarily advanced garden symbionts of higher attine ants, but not by the fungi reared by the basal lineages of this ant clade.

**Conclusions:**

Pectinolytic enzymes produced in the gongylidia of the fungal symbiont are ingested but not digested by *Acromyrmex *leaf-cutting ants so that they end up in the fecal fluid and become mixed with new garden substrate. Substantial quantities of pectinolytic enzymes are typically found in pathogenic fungi that attack live plant tissue, where they are known to breach the cell walls to allow the fungal mycelium access to the cell contents. As the leaf-cutting ant symbionts are derived from fungal clades that decompose dead plant material, our results suggest that their pectinolytic enzymes represent secondarily evolved adaptations that are convergent to those normally found in phytopathogens.

## Background

The fascinating natural history of obligate mutualistic symbioses, in which two completely different organisms live in a close and fully interdependent relationship, are increasingly unraveled by studies of extant adaptations and phylogenetic history [1-3]. Such intimate relationships have the potential to become very complex because their coevolution may produce specialized structures that provide indirect fitness via the symbiotic partner [4,5]. This may constrain a detailed understanding of the key molecular processes that maintain the symbiosis, so that identifying the gene sequences involved in these processes is of paramount importance. New high-throughput methods offer the opportunity to obtain a more comprehensive molecular understanding of so-called "nonmodel" organisms involved in obligate symbioses. Illustrative examples are the recent genome sequencing projects on *Laccaria *and black truffle fungi, which have led to significant new insights into the respective roles of these fungi in ectomycorrhizal symbioses [6,7].

Leaf-cutting ants of the genera *Acromyrmex *and *Atta *live in mutualistic symbiosis with a basidiomycete fungus (*Leucocoprinus gongylophorus*), which they cultivate as fungal gardens in underground nest chambers. The ants provide the fungus with a growth substrate consisting of freshly cut leaf fragments. After new leaf fragments are brought into the nest, the ants chew them into smaller pieces and apply droplets of fecal fluid to the leaf pulp before depositing this mixed substrate in the fungus garden and inoculating it with small tufts of mycelium from older parts of the garden [8]. Previous work has shown that the fecal fluid of a wide range of attine species contains various digestive enzymes such as proteases, amylases and chitinases [9-12]. The fecal fluid of *Atta colombica tonsipes *has also been shown to contain enzymes to degrade pectin, xylan, carboxymethylcellulose and several disaccharides as well as synthetic *p*-nitrophenyl glycosides [13]. It has been suggested that the three fecal fluid proteases of *Atta texana *and *Atta colombica tonsipes *originate from the fungal symbiont, as proteases with similar biochemical properties could be isolated from the garden mycelium [14]. A more recent study used isoelectric focusing to show that cellulases, pectinases and laccases present in the fecal droplets of *Acromyrmex *leaf-cutting ants had properties similar to those of fungus-produced enzymes, which likewise suggested a fungal origin of these enzymes [15]. However, it is rarely made explicit that the implications of this notion are rather spectacular, as this would imply that these fecal proteins are not affected by the digestive system of the ants and that they must have important functions for the symbiosis to compensate for the ants not under selection to digest these proteins.

The primary food source of leaf-cutting ants consists of the swollen hyphal tips, called gongylidia, that their fungus gardens produce in clusters (staphylae). These structures are not known from any other fungi and are believed to be produced exclusively for the benefit of the ants. If the fecal droplet proteins would indeed be directly derived from the fungus ingested by the ants, we should expect the gongylidia to be the primary source of these proteins. Here we use state-of-the-art molecular techniques (sodium dodecyl sulfate-polyacrylamide gel electrophoresis (SDS-PAGE), matrix-assisted laser desorption ionization time of flight (MALDI-TOF) mass spectrometry, quantitative polymerase chain reaction (qPCR), gene cloning, and quantitative enzyme assays) to test the validity of the idea that fungal proteins are excreted in ant fecal droplets to be recycled for active service in the decomposition of new leaf fragments.

Our study focuses on pectinolytic enzymes, as previous studies have shown that these enzymes are present in the ant fecal droplets and because pectinolytic enzymes are known to play a key role in the infection process of live plants by necrotrophic fungal pathogens [16-18]. The fungi that are cultivated by leaf-cutting ants of the genera *Acromyrmex *and *Atta *belong to a clade of universal decomposers (Basidiomycotina: Agaricaceae: Leucocoprinae), but secondarily, and exceptionally, have evolved the ability to decompose live plant material that is offered to them as substrate by the farming ants [19]. The ant-fungus mutualism is thus facing similar selection pressures as necrotrophic fungi, that is, to breach the cell walls of living plant tissues to gain access to the nutritious interior of these cells. We therefore tested the hypothesis that gongylidia consumption and fecal droplet deposition might have evolved to concentrate and transport fungal pectinolytic enzymes to the upper part of fungus gardens to enhance the degradation of live plant cell walls immediately after they are processed by the ants.

We confirm that a large number of distinct proteins survive gut passage, that many of them are pectinolytic enzymes, that they are active in fecal droplets only when the ants are allowed to feed on symbiont mycelium and that the genes encoding these proteins are overexpressed in the gongylidia.

## Results

**Figure 1:**
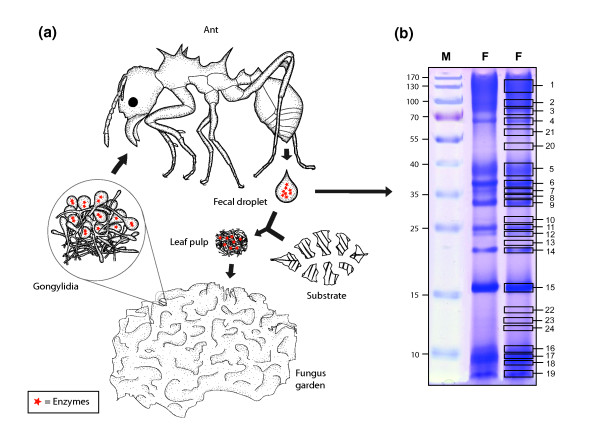
**The life cycle of fecal droplet proteins. **(a) Degradation enzymes produced in the gongylidia are passed through the ant alimentary system to end up in the fecal fluid, fecal droplets of which are mixed with the new plant substrate to form leaf pulp that the ants add to the fungus garden. **(b) **Sodium dodecyl sulfate-polyacrylamide gel electrophoresis (SDS-PAGE) of ant fecal fluid. M, molecular size marker. F, fecal fluid. Excised bands are indicated by numbered boxes in the right-hand version of duplicated lane F.

SDS-PAGE of fecal droplet proteins resulted in a specific pattern of protein bands (Figure 1b) that was highly reproducible. To identify the proteins, 24 bands were extracted from the gel and subjected to tandem mass spectrometry (MS/MS). This resulted in 90 different peptide sequences determined from the MS/MS spectra. In a first approach to identifying the proteins from which these peptides originated, we designed degenerate primers matching the amino acid sequences of the peptides and used them in PCR reactions using cDNA from the fungal symbiont as template. By this method, we were able to identify 14 fecal droplet proteins. In a second approach, the sequences were used in a Basic Local Alignment Search Tool (BLAST) search of a newly generated database (M. Schiøtt *et al.*, unpublished work) containing approximately 700 Mb of genome sequence data of the fungal symbiont obtained by 454 sequencing [20], which led to the identification of an additional 19 fecal droplet proteins. In both cases, rapid amplification of cDNA ends (RACE)-PCR was subsequently used to obtain the full-length sequences of the genes encoding these proteins. Among the 33 gene sequences identified by these methods, 7 were found to share a high degree of similarity with enzymes involved in the degradation of pectin (Table 1). The molecular mass of the proteins calculated from the corresponding cDNA sequences were in all cases similar to the molecular mass predicted from SDS-PAGE, indicating that the proteins in the fecal material are full-length proteins and not just short degradation products.

**Table 1:** Protein sequences of pectinolytic enzymes in ant fecal fluid and corresponding molecular weights in daltons

Protein	**Mass spectrometry data^a^**	Sequence data	Band^b^	MW (Da)
Arabinofuranosidase	FPGGNNJENTVDQR	FPGGNNLENTVDQR	4	69139
	GD[YQ/QY]JPSTJPSSTGTVFWSVVR	GDQYIPSTLPSSTGTVFWSVVR		
	NDJASAJAEVG[SP/PS]FWR	NDIASALAEVGPSFWR		
	PEDFAANTYTFR	NEDFFAANTYTFR		
	STAJPNAJHVVJPTGR	STALPNALHVVIPTGR		
	VESAAGEAAFMTGJER	VESAAGEAAFMTGLER		
	YFE[W/GE]YAAJSTNNP/PNDJFG	YFEGEYAAISTNPNDIFG		
Endogalactanase	DJDGJNTQJFTYTR	DLDGLNTQIFTYTR	7	37416
	GAVTPFEEJJHNHGA[J/N]	GAVTPFEELLHNHGAN		
	GWFSSJANJESSGR	GADFSSLANLESSGR		
Pectin esterase	GQAYFGGNTJ[QR/GVK]	GQAYFGGNTLGVK	6 + 7 + 8 + 9 + 10 + 18 + 19 + 22	37571
	GAGWVTASGR	GAGWVTASGR		
	GYJEGATDFJFGQR	GYIEGATDFIFGQR		
	NNQATJQFGJDAGQAGSDDASGTJR	NNQATIQFGLDAGQAGSDDASGTLR		
	NTFGVGSQAJAJSQYGDR	NTFGVGSQAIALSQYGDR		
	QAYFGGNTJGVK	QAYFGGNTIGVK		
Pectate lyase	JVJJSGNJSGDAVVR	IVLLSGNLSGDAVVR	7 + 8	
	VJNENNVJJR	VLNENNVIIR		
Polygalacturonase	VAVN(1710)...(1206)TGTWNWSNJK	VAVNCGVGSCTGTWNWSNLK	6	37055
Rhamnogalacturonan acetylesterase-1	PDNJWVNGEJGAGPR	PDNIWVNGEIGAGPR	10 + 16 + 17 + 18 + 19	26946
	FVGYAQTAASR	FVGYAQTAASR		
	VNDAJAGR	VNDAIAGR		
Rhamnogalacturonan acetylesterase-2	FVTYAQSJGJR	FVTYAQSLGLR	18	26467

^a^The letter J symbolizes the amino acids leucin and isoleucin, which have the same molecular mass and therefore are indiscernible by mass spectrometry. ^b^These numbers refer to the bands excised from the SDS-PAGE gel (Figure 1) in which the protein in question was found.

**Figure 2:**
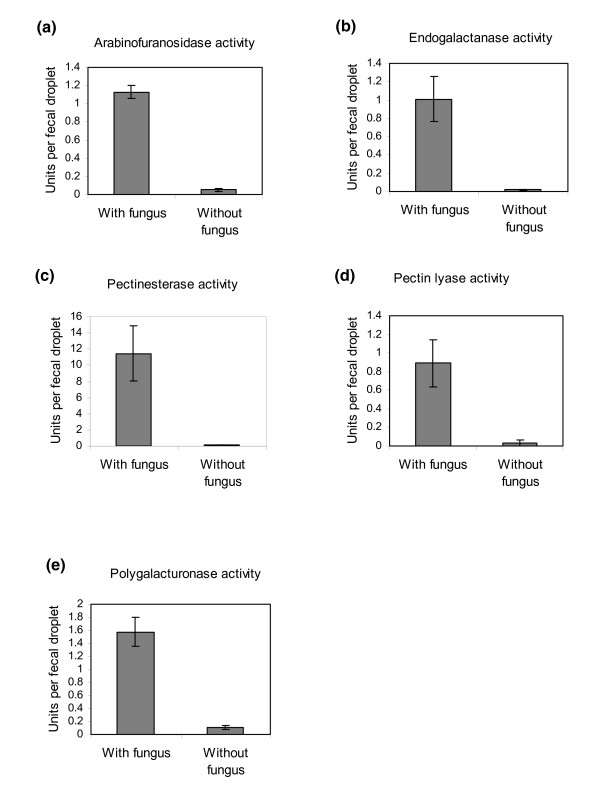
**Pectinolytic enzyme activities in fecal material from ants with access to fungal symbiont food compared to ants deprived of their fungus garden material for 2 weeks**. Bars are means ± SE (n = 9).

To reveal whether the obtained pectinolytic enzymes were in fact active in the fecal droplets of the ants, activity assays of five of the six identified types of enzymes were performed on single fecal droplets of nine ants taken from each of three different colonies. The same activity measurements were performed on fecal material from a similar number of ants that were kept for more than 2 weeks on sugar water and bramble leaves, but without the fungal symbiont to confirm the fungal origin of the enzyme activities. Without exception, the enzyme assays showed pronounced activity in fecal material from ants kept with their fungal symbiont, whereas fecal material from ants deprived of their symbiont showed almost no activity (Figure 2). The low activities apparently still present in some of the assays on fecal droplets from the starved ants were most likely artefacts because any colored substances in the fecal material will absorb light during the spectrophotometric assays and will consequently produce a weak false-positive signal. Alternatively, some enzyme activity may remain in the fecal fluid of the ants even after 2 weeks of separation from the fungal symbiont. Enzyme assays of heat-denatured fecal droplets hardly showed any activity, confirming the appropriateness of our activity measurements (data not shown). Activity measurements for the sixth pectinase, rhamnogalacuronan acetylesterase, were not been performed as no suitable assay could be found.

**Figure 3:**
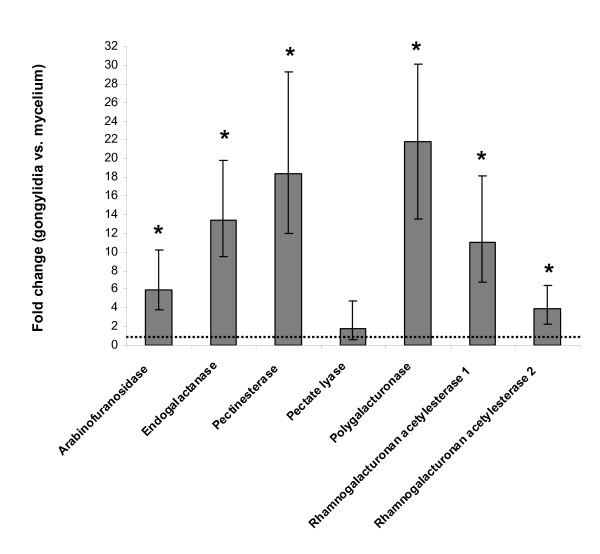
**Expression levels of pectinolytic enzyme genes in gongylidia relative to gongylidia-free mycelium measured with quantitative real-time polymerase chain reaction (qPCR)**. Bars are means ± SE (n = 4). Statistically significant results are marked with an asterisk. An equal level of gene expression between the two tissues gives a fold change of one (dotted horizontal line).

To test whether the expression level of the identified pectinolytic enzymes is upregulated in the gongylidia, the main food of the ants, RNA was extracted both from gongylidia and from gongylidia-free mycelium taken from fungus gardens of four different colonies. After reverse transcription of the mRNA into cDNA, the expression levels of the genes were measured by qPCR using three different reference genes to normalize the data. Six of the seven genes showed significant upregulation in the gongylidia (Figure 3), while one gene (pectate lyase) was equally expressed in the two types of tissue. The latter gene had a relatively high cycle threshold (C_t_) value in the qPCR (and also a high PCR efficiency value), suggesting that it was only weakly expressed. Peptide sequence tags for the pectate lyase genes were found only in the weak bands 7 and 8 (Figure 1b), confirming that this enzyme does not play an important functional role in the fecal droplets.

## Discussion

Our tandem mass spectrometry analyses identified seven pectinolytic enzymes in the fecal material of *Acromyrmex echinatior *leaf-cutting ants. Activity assays showed that these enzymes were still active after passage through the ant gut, and our feeding and gene cloning experiments showed that these enzymes must be derived from the ingested fungal tissue (Figure 1a). This is consistent with findings that pectinolytic enzymes are very rarely produced by animals [21,22]. The number of proteins that we were able to visualize on the SDS-PAGE gels remained limited (Figure 1b), suggesting that many other proteins in the fungal food are being degraded in the ant guts. This is as expected because proteins are valuable nutrients with high nitrogen content, a resource that is clearly limiting for the overall performance of the symbiosis [23,24]. The fact that we were able to document the earlier inference [14,15] that some proteins, including pectinolytic enzymes, are not digested in the ant gut indicates that selection for decomposition efficiency has affected the exosymbiosis between ant and fungi at a deep molecular level. We expect that other bands on our SDS-PAGE gels represent proteins with important functions in the symbiosis, and work to unravel these functions is ongoing.

### The production, circulation and function of pectinolytic enzymes in the symbiosis

Gongylidia, the swollen hyphal tips produced by the fungal symbionts of the higher attine ants (including the leaf-cutting ants), are generally considered to be the almost exclusive food source of the ants and their broods [8,25-27]. They are found nowhere else other than in these fungal symbionts and thus represent an evolutionarily derived trait that has been inferred to be adaptive for symbiosis [27]. However, the details of this adaptive function have remained poorly understood. For example, it has previously been suggested that only the larvae of leaf-cutting ants rely exclusively on a fungal diet [28], whereas later research has shown that adult workers also ingest significant amounts of fungus garden material [29], a notion that is corroborated by our present study. Here we have shown that the gongylidia not only serve as nutrition for the ants but also produce enzymes needed for the degradation of the plant substrate in much higher quantities than in undifferentiated mycelium. Our results thus imply that we have likely discovered an important adaptive function of the gongylidia, as we showed earlier that pectin degradation in newly grown sections of leaf-cutting ant gardens proceeds at a very high rate and with the primary function of providing access to the fresh cell contents of the leaf tissue [23]. The farming ants clearly prioritize highly digestible nutrients such as proteins and starch relative to the bulk of cell wall material, which tends to be discarded on the dump piles that leaf-cutting ants maintain [23,25] (Møller I, De Fine Licht HH, Harholt J, Willats WGT, Boomsma JJ: The dynamics of plant cell-wall polysaccharide decomposition in leaf-cutting ant fungus gardens, unpublished work). These studies also indicate that the degradation of pectin itself is unlikely to contribute much to the available resources of a leaf-cutting ant nest relative to the proteins and starch from the interior of the plant cells.

Pectinolytic enzymes are known to be important for the virulence of both bacterial and fungal phytopathogens [17]. A recent study comparing the gene inventories of phytopathogenic versus free-living filamentous ascomycetes found that pectinolytic enzyme genes were more common in pathogenic fungi [18]. When the pathogenic fungus *Fusarium oxysporum *is grown on a plant cell wall substrate, pectinolytic enzymes are the first enzymes to be secreted, followed by other carbohydrate active enzymes [30]. Gene disruption studies have shown that the virulence of fungal phytopathogens may decrease significantly when a pectinolytic enzyme gene is mutated [31], although in most cases more than one gene has to be mutated to affect virulence, owing to redundancy between multiple isoforms of these genes in the fungal genomes [32]. Also, *Erwinia chrysanthemi *bacteria produce a wide range of pectinolytic enzymes upon infection of plant tissue, and inactivation of at least some of these genes makes infection by these bacteria ineffective.

Plant pathogenic microorganisms typically produce many different pectinolytic enzymes targeting both the pectin side chains and the backbones of the molecules, as both are apparently needed to breach the cell wall to gain access to the cell content [16,17]. In our study, we found one pectinolytic enzyme targeted toward the backbone of the smooth region that was upregulated in the gongylidia (polygalacturonase), whereas another (pectate lyase) was found without being upregulated (Figure 3). Otherwise, we found a pectinesterase that removes methyl groups of the smooth region of pectin molecules and several enzymes targeted toward the hairy regions, including an arabinogalactan endo-1,4-β-galactosidase and an arabinofuranosidase that, respectively, remove galactose and arabinose from the side chains. In addition, we found two very similar rhamnogalacturonan acetyl esterases, which remove acetyl groups from the rhamnose moieties of the hairy region. However, no degradation enzymes targeted toward the backbone of the pectin hairy regions were found. We have previously found enzymatic activity toward rhamnogalacturonan in the fecal droplet material (unpublished work Schiøtt M), but apparently the abundance of that protein was not high enough to be detected by the methods used in the present paper.

### Selection pressure, evolutionary pathways and convergence

When new leaf material is brought into the ant nest, it is added to the top of the fungus garden, and tufts of mycelium are subsequently placed on it [8]. This means that the leaf-to-mycelium ratio in the top of gardens is very high, so that production of digestive enzymes by these small mycelial tufts is likely to be a limiting step in the degradation process. Application of high concentrations of these enzymes via the fecal fluid of the ants thus increases the turnover rate of garden substrate, which may provide some compensation for the relatively low nitrogen to carbon ratio of the leaf material. This may be why this intricate pathway of having pectinolytic enzymes brought in from mature parts of the garden via the digestive system of the ants could evolve by favoring mutations that allowed these enzymes to accumulate in the hindgut rather than being degraded.

Plant tissues usually contain chemical compounds that have evolved to protect against attack from herbivores and fungal or bacterial pathogens [33]. Being of saprotrophic origin, the free-living ancestors of the attine ant fungal symbionts may not have possessed mechanisms to cope with such defenses expressed in live plant tissues. The chewing behavior of ant workers turning leaf fragments into pulp before mycelium is inoculated may therefore make decomposition highly effective when it is combined with the application of large amounts of pectinolytic enzymes that open up and kill the plant cells so that the production of any secondary defensive compounds is terminated. This would imply that it is the combined effort of the unholy alliance [34] between ants and fungus that allowed the efficient utilization of fresh leaves as a novel resource, something neither of the symbiotic partners would have been able to do on its own.

The fungi that are cultivated by Latin American leaf-cutting ants of the genera *Acromyrmex *and *Atta *are highly peculiar, because they belong to a universally dead substrate decomposition clade (Agaricales: Agaricaceae: Leucocoprinae), but have secondarily evolved the ability to degrade live plant material when it is offered to them as substrate by the ant farmers [19]. Recent studies have shown that the fungus reared by the leaf-cutting ants throughout their range may be a single genetically variable species (*Leucocoprinus gongylophorus*) that replaced older symbionts in a single selective sweep only a few million years ago [35]. Future studies should address the extent to which pectinolytic enzymes are upregulated in the gongylidia of the less derived fungal symbionts of *Trachymyrmex *and *Sericomyrmex*, genera of higher attine ants that also rear symbionts with gongylidia, but with a much lower proportion of fresh leaf material in their diet [36]. In addition, it would be highly interesting to unravel the degree of convergence at the molecular level between the pectinolytic enzymes of leaf-cutting ant garden symbionts and fungi that are free-living parasites of fresh leaves.

## Conclusions

We have shown that pectinolytic enzyme genes are overproduced in the gongylidia of leaf-cutting ant fungal symbionts. After ingestion of the gongylidia, the enzymes pass through the ant gut and end up in the fecal fluid, where a significant fraction of these enzymes remain functional. Droplets of this fecal fluid are mixed with the new leaf material that the ants fragment to become fungus garden substrate, and we infer that it is particularly in this time window that the pectinolytic enzymes enhance the efficiency of the symbiosis. This jointly produced adaptive response to overcome cell wall defenses of fresh plant substrate resembles the enzymatic mechanisms that phytopathogenic fungi and bacteria realize by themselves when facing similar challenges of decomposing fresh plant material. This suggests either that these traits have evolved convergently in the different fungal lineages or that the ancestors of the leaf-cutting ant symbionts were somehow preadapted to a necrotrophic lifestyle. We believe that the former explanation is most likely, but explicit studies on nondomesticated leucocoprinous fungi are needed to confirm this contention.

## Methods

### Biological material and a summary of pectin cell biology

Colonies of *Acromyrmex echinatior *(numbers Ae263, Ae280, Ae282, Ae322, Ae332, Ae335 and Ae349) were collected in Gamboa, Panama, in 2004-2007 and maintained in the laboratory under standard conditions of about 25°C and about 70% relative humidity [37], where they were supplied with a diet of bramble leaves, rice and pieces of apple. In experiments using ants that were deprived of their fungal symbiont, the ants were instead fed a diet of bramble leaves and 20% sugar water. Fecal droplets were collected by squeezing large worker ants with forceps on the head and abdomen until they deposited a drop of fecal material. A quantity of 0.5 μL of water was added to the droplet before it was collected with a micropipette.

Pectins are polysaccharides composed of smooth regions of (1,4)-linked α-D-galacturonic acid (Gal*p*A) interspersed with hairy regions where (1,2)-linked α-L-rhamnose alternates with Gal*p*A [38,39]. About half of the rhamnose residues are branched with mainly L-arabinosyl- and/or D-galactosyl-containing side chains, although several other types of sugars may also be used [40]. Breakdown of the backbone of the smooth regions is accomplished by endo- and exopolygalacturonases, as well as by pectin and pectate lyases, whereas breakdown of the backbone of the hairy regions is mediated by rhamnogalacturonan hydrolase, rhamnogalacturonan galacturonohydrolase, α-rhamnosidase and rhamnogalacturonan lyase [41]. A wide variety of enzymes related to release of L-arabinose and D-galactose are also used to break down the side chains of the hairy regions [41]. Finally, the activity of these pectinolytic enzymes depends on the degree of methylation and acetylation of the pectin, so that the removal of methyl and acetyl groups by methyl and acetyl esterases is also a prerequisite for efficient pectin degradation. This general knowledge of pectin biochemistry allowed us to make some inferences with regard to the functions of the pectinolytic enzymes that we were able to identify.

### SDS-PAGE and mass spectrometry

Fifty fecal droplets from workers of colony Ae263 were collected and put immediately into 2× SDS-PAGE loading buffer (100 mM Tris·HCl, pH 6.8, 200 mM dithiothreitol, 4% SDS, 0.2% bromophenol blue, 20% glycerol) and loaded onto a 12.5% polyacrylamide gel [42]. The gel was run for 1 hour at 60 mA, followed by 1 hour at 75 mA with cooling, and stained for 3 hours with Coomassie Brilliant Blue R250 (0.25% Coomassie Brilliant Blue R250, 0.44% ethanol, 9.2% acetic acid) and destained in 5% ethanol and 7.5% acetic acid overnight.

Sample preparation methods for MS were modified from Shevchenko *et al. *[43]. In brief, bands of interest were manually excised from the gel and washed with deionized water followed by two washes with 100% acetonitrile for 15 and 2 minutes, respectively. The gel plugs were dehydrated in a vacuum centrifuge and rehydrated with a solution of 2% trypsin (Promega, Madison, WI, USA) in 50 mM NH_4_HCO_3 _at 4°C. After 20 minutes, the excess of trypsin solution was removed and 30 μL of 50 mM NH_4_HCO_3 _were added to allow digestion to proceed at 37°C overnight, after which samples were stored at -20°C until use. Peptide desalting was performed on custom-made reverse-phase microcolumns prepared with R2 resin (Perseptive Biosystems Inc., Framingham, MA, USA) as described elsewhere [44]. Peptide solutions obtained from digestions of each separate spot were loaded onto a microcolumn, followed by washing with 10 μL of 1% trifluoroacetic acid (TFA). Bound peptides were eluted with 0.8 μL of matrix solution (5 μg/μL α-cyano-4-hydrocynnamic acid in 70% acetonitrile and 0.1% TFA) directly onto the matrix-assisted laser desorption ionization (MALDI) target plate. Peptide mass spectra were acquired in positive reflector mode on a 4800 Plus MALDI-TOF/TOF™ Analyzer (Applied Biosystems, Foster City, CA, USA) using 20 kV of acceleration voltage. Each spectrum was obtained with a total of 800 laser shots and was externally calibrated using peptides derived by tryptic digestion of β-lactoglobulin.

Tandem mass spectra were acquired using the same instrument in MS/MS-positive mode. From the raw data output, peak lists were generated using Data Explorer (Applied Biosystems, Foster City, CA, USA). MS and MS/MS peak lists were combined into search files and used to search the National Center for Biotechnology Information protein sequence database using the Mascot search engine (Matrix Science Ltd, London, UK). Since sequence information was not available in the database from either the ant or the fungus, the searches did not result in identification of any protein. As a consequence, manual *de novo *sequencing was performed on the basis of the presence of b and y peptide fragment ions in MS/MS spectra [45]. To facilitate *de novo *sequencing, the remaining sample was derived by adding 7 μL of 10 μg/μL 4-sulfophenyl isothiocyanate dissolved in 50 mM NaHCO_3_, pH 8.6. The reaction was allowed to proceed for 30 minutes at 50°C and terminated using 1 μL of 1% TFA. The mixture was then loaded on a desalting column (as described above), eluted on the target and analyzed using 4800 Proteomics Analyzer (Applied Biosystems, Foster City, CA, USA) in MS/MS mode. Derived peptides (showing a mass difference of 215 Da compared to the original MS spectra) were sequenced using the same instrument in MS/MS-positive mode. The obtained MS/MS spectra from underivatized and derivatized samples were analyzed manually supported by the AminoCalc program (Protana A/S, Odense, Denmark) to find the distance between fragment ions and to obtain amino acid sequences [46].

### RNA isolation

RNA was isolated from the fungal symbiont using an RNeasy Plant Mini Kit (Qiagen, Hilden, Germany) with modifications of the protocol. Fungus garden material was ground in liquid nitrogen and 100 mg were added to 700 μL of the RLC lysis buffer (with addition of β-mercaptoethanol) included in the RNeasy Plant Mini Kit (Qiagen, Hilden, Germany). The RNA was extracted twice with an equal amount of phenol/chloroform/isoamyl alcohol (25:24:1), pH 8, followed by one extraction with chloroform/isoamyl alcohol (24:1). The final extract was further purified using the RNeasy Plant Mini Kit following the enclosed protocol from the step where the extract is loaded on a QIAshredder column. An on-column DNase I treatment step was included as described in the protocol.

### PCR and gene cloning

Sequencing of full length gene products was performed using a RACE strategy. 3"- and 5"-RACE libraries were made from approximately 1 μg of the purified RNA with the SMART RACE cDNA kit (Clontech, Mountain View, CA, USA), and gene sequences were PCR amplified from these libraries using specific primers designed from the fungal genome sequence along with the primers enclosed in the SMART RACE cDNA kit (for primer sequences, see Additional file 1, Table S1). The following PCR scheme was used to amplify the genes: one cycle of 95°C for 5 minutes, 10 cycles of 94°C for 20 seconds, 72°C for 30 seconds (with a decrease in temperature of 0.5°C in every cycle) and 72°C for 2 minutes, followed by 35 cycles of 94°C for 20 seconds, 67°C for 30 seconds and 72°C for 2 minutes, and ending with one cycle of 72°C for 7 minutes. Five of the pectinolytic genes were initially cloned by degenerate PCR using the cDNA libraries described above as the template. In these instances, the PCR scheme used was as follows: one cycle of 95°C for 5 minutes, 35 cycles of 94°C for 20 seconds, 50°C for 30 seconds and 72°C for 2 minutes, and ending with one cycle of 72°C for 7 minutes. All PCR products were cloned in pCR4-TOPO before sequencing using the TOPO TA cloning method (Invitrogen, Carlsbad, CA, USA). Gene sequences were deposited in GenBank with the accession numbers HQ174763-HQ174771. For primer sequences, see Addtional file 1, Table S1.

### Quantitative real-time PCR

Pure mycelium and staphylae (clusters of gongylidia) used for real-time PCR were collected with forceps from small pieces of fungus garden (colonies Ae263, Ae280, Ae322, Ae335) using a stereomicroscope and put directly into liquid nitrogen before further processing to extract RNA (see RNA isolation section above). Gene expression levels of the pectinolytic enzymes in gongylidia and gongylidia-free mycelium were determined using qPCR. Either 200 ng or 1,000 ng of total RNA from pure mycelium or staphylae (clusters of gongylidia) were reverse-transcribed to cDNA with Superscript III reverse transcriptase (Invitrogen) and an oligo(dT) primer and subsequently diluted 40 times with water. cDNA (0.5 μL) was used in a 20-μL qPCR reaction with 10 μL of 2× SYBR Premix Ex Taq (TaKaRa Bio Inc., Otsu, Japan) and 0.4 μl of each primer (10 μM). The qPCR was run on a Mx3000P QPCR system (Agilent, Santa Clara, CA, USA) with PCR conditions consisting of one cycle of 95°C for 2 minutes, then 40 cycles of 95°C for 30 seconds, 55°C for 30 seconds and 72°C for 30 seconds. A melt curve was included after each run. The primers used in the qPCR procedure were all positioned in the 3" end of genes and amplified a DNA fragment of about 250 bps. At least one of the primers in a pair was spanning an intron to prevent amplification of genomic DNA (for primer sequences see Additional file 1, Table S1). qPCR reactions were run in triplicate, and the mean C_t _value was used in the subsequent analyses. The transcript levels were normalized using three different reference genes: Elongation factor 1-α (GenBank HQ191273), ubiquitin (GenBank HQ174771) and glyceraldehyde 3-phosphate dehydrogenase (GenBank HQ174770). The efficiency of the qPCR reactions was measured using a dilution series of templates at four different concentrations. The relative gene expression levels between mycelium and gongylidia were determined using the software program REST (Qiagen, Hilden, Germany) [47], which uses a pairwise fixed reallocation randomization test for assessing the significance of the obtained values.

### Enzyme assays

Enzyme activities were measured in fecal droplets harvested from colonies Ae280, Ae332 and Ae349. Arabinofuranosidase activity was determined by incubating one fecal droplet in 100 μL of 1 mM 4-nitrophenyl α-L-arabinofuranoside (Sigma N3641) (Sigma-Aldrich, St. Louis, MO, USA) and 50 mM sodium acetate, pH 5, for 60 minutes at 30°C. A quantity of 100 μL of 0.5 M Na_2_CO_3 _were added, and the absorbance was read in a Versamax Plus plate reader (Molecular Devices, Sunnyvale, CA, USA) at 405 nm. The absorbance measurements were converted to enzyme units using a standard curve made with 4-nitrophenol (Sigma 241326). One unit was defined as the amount of enzyme able to release 1 nM nitrophenol per minute.

Endogalactanase activity was determined by incubating one fecal droplet in 50 μL of 1% Azo-galactan (Megazyme International Ireland Ltd., Bray, Ireland) and 50 mM sodium acetate, pH 5, for 60 minutes at 30°C. A quantity of 125 μL of 96% ethanol was added, and the mixture was incubated for 10 minutes before being centrifugated at 1,000 *g *for 10 minutes. A quantity of 150 μL was transferred to a microtiter plate, and the absorbance was read in a plate reader at 590 nm (Versamax Plus). The absorbance measurements were converted to enzyme units using the standard curve provided by the manufacturer. One unit was defined as the amount of enzyme able to release 1 nM galactose equivalents per minute.

Pectate lyase activity was determined by incubating one fecal droplet in 50 μL of 0.5% citrus pectin (Sigma P9135), 50 mM TrisÂ·HCl buffer, pH 7, 1 mM CaCl_2 _for 30 minutes at 30°C. The absorbance at 230 nm was read before and after incubation. One unit was defined as the amount of enzyme able to increase the absorbance by one unit per hour in a 1-cm path length.

Polygalacturonase activity was determined by incubating one fecal droplet in 100 μL of 0.5% polygalacturonic acid (Sigma P3889), 50 mM sodium acetate, pH 5, for 10 minutes at 30°C. A quantity of 50 μL of DNS solution (0.4 M NaOH, 0.04 M 3,5-dinitrosalicylic acid, 1 M potassium sodium tartrate) was added, and the mixture was heated at 99.9°C for 5 minutes in a PCR machine [48]. A quantity of 50 μL of the mixture was added to 150 μL of water, and the absorbance at 540 nm was read in a plate reader (Versamax Plus). The absorbance measurements were converted to units using a standard curve made with glucose. One unit was defined as the amount of enzyme able to release 1 μg of glucose equivalents per minute.

Pectinesterase activity was determined using a titrimetric method [49]. One fecal droplet was added to 250 μL of 1% citrus pectin (Sigma P9135), 100 mM NaCl, pH 6.1, and pH was maintained at 6.1 over a 5-minute period by addition of 5 mM NaOH. The amount of NaOH used corresponds to the amount of protons released by the activity of pectinesterase, and one unit of enzyme activity was consequently expressed as the nanomolar NaOH added per minute.

## Abbreviations

cDNA: complementary deoxyribonucleic acid; Da: dalton; DNS: 3,5-dinitrosalicylic acid; Gal*p*A: (1,4)-linked α-D-galacturonic acid; MALDI-TOF: matrix-assisted laser desorption/ionization-time of flight; MS: mass spectrometry; MS/MS: tandem mass spectrometry; MW: molecular weight; qPCR: quantitative polymerase chain reaction; RACE-PCR: rapid amplification of cDNA ends-polymerase chain reaction; SDS-PAGE: sodium dodecyl sulfate-polyacrylamide gel electrophoresis; TFA: trifluoroacetic acid.

## Competing interests

The authors declare that they have no competing interests.

## Authors' contributions

MS and JJB designed the study. MS performed the experimental work except for the mass spectrometry. ARW and PR designed and performed the mass spectrometry. MS and JJB wrote the paper with input from ARW and PR. All authors read and approved the final manuscript.

## Supplementary Material

Additional file 1 **Table S1**. List of primers used in the study. Click here for file
