# Supplementary material for: Leaf-cutting ant fungi produce cell wall degrading pectinase complexes reminiscent of phytopathogenic fungi
Source: BMC Biol. 2010 Dec 31;8:156. doi: 10.1186/1741-7007-8-156 (PMC3022778; doi:10.1186/1741-7007-8-156)
Supplement: Additional file 1 — Table S1. List of primers used in the study. [file 1741-7007-8-156-S1.doc]

Table S1. List of primers used in the study.

| Gene | Primer function | Primer sequences |
| --- | --- | --- |
| Arabinofuranosidase | Forward degenerate primer | 5’-GGTGARGCNGCNTTYTTYAC-3’ |
|  | Reverse degenerate primer | 5’-CACCARAAGACNGTNCCNGT-3’ |
|  | 5’ RACE primers | 5’-GCCGGAAACGTTTACCGGTCAACG-3’ |
|  | 3’ RACE primer | 5’-GCCCGAGAAGAGGTGCATAGGATG-3’ |
|  | Forward qPCR primer | 5’- ATGCACCTCTTCTCGGGCAT-3’ |
|  | Reverse qPCR primer | 5’-CTGCCCGTGTTAGAAATCTTG-3’ |
| Endogalactanase | Forward degenerate primer | 5’-GCTGTNACNCCXTTYGARGA-3’ |
|  | Reverse degenerate primer | 5’-TARGTGAAVADYTGXGTRTT-3’ |
|  | 5’ RACE primers | 5’-GCCCGTCGGACGTGCTCGTCC-3’  5’-GCGAGGTTTGCACCGTGGTTATGCAAG-3’ |
|  | 3’ RACE primer | 5’-TGGCCCAAAGACCTCGATGGCCTC-3’ |
|  | Forward qPCR primer | 5’-TTGTCAACAAATTTGGGAAGGATG-3’ |
|  | Reverse qPCR primer | 5’-GACGAGCAGGTTGTCGGTG-3’ |
| Pectin esterase | Forward degenerate primer | 5’-CTTGAYGCNGGXCARGCNGG-3’ |
|  | Reverse degenerate primer | 5’-TGACCRAADATRAARTCNGT-3’ |
|  | 5’ RACE primers | 5’-CCACTTGCATCGTCGGAACCGGC-3’ |
|  | 3’ RACE primer | 5’-GTGCTCAGGTCTACCTGCGAGGC-3’ |
|  | Forward qPCR primer | 5’-TTGGACGACCATGGAGCAATT-3’ |
|  | Reverse qPCR primer | 5’-TGCTTGAGAAGCTGAAAGGAC-3’ |
| Pectate lyase | 5’ RACE primers | 5’-GAGGGTTGCATAGCCAACATCAGCAAC-3’ |
|  | 3’ RACE primer | 5’-GCCGGCGTCATTCAAAAACGTGCTTC-3’ |
|  | Forward qPCR primer | 5’-GAATAACGTTTGGACTGGCAC-3’ |
|  | Reverse qPCR primer | 5’-GGAATTGATATCAGAAGGACAGG-3’ |
| Polygalacturonase | Forward degenerate primer | 5’-CAYAAYACTGAYGGNTTYGA-3’ |
|  | Reverse degenerate primer | 5’-GTCCARTTCCANGTNCCNGT-3’ |
|  | 5’ RACE primers | 5’-CCATGACCACCAGAGCAGTGGTTG-3’ |
|  | 3’ RACE primer | 5’-CTGCCTTGCCATCAATCGTGGTGC-3’ |
|  | Forward qPCR primer | 5’-GTGTCATCATTGACCAGAGCT-3’ |
|  | Reverse qPCR primer | 5’-CCAGAGTATACAATGTAGTAGATTG-3’ |
| Rhamnoglacturonan acetylesterase-1 | Forward degenerate primer | 5’-TGGTTYGGNCAYAAYGAYGG-3’ |
|  | Reverse degenerate primer | 5’-GTTTGAGCRTANCCNACRAA-3’ |
|  | 5’ RACE primers | 5’-CGGGGGTCTGAGAAGATACGATAGG-3’ |
|  | 3’ RACE primer | 5’-CGCCTTCGGGGATGGATACGAC-3’ |
|  | Forward qPCR primer | 5’-CTGTGACTAATGCCGCGGG-3’ |
|  | Reverse qPCR primer | 5’-TCTGTCCCAGTGCATCATATG-3’ |
| Rhamnoglacturonan acetylesterase-2 | 5’ RACE primers | 5’-GTACCACCGCCACCTCCACCTAC-3’ |
|  | 3’ RACE primer | 5’-CGTAAGGCTTGCTGGCGATTCCAC-3’ |
|  | Forward qPCR primer | 5’-GTCACAACCGCAACCGGT-3’ |
|  | Reverse qPCR primer | 5’-CACCAAGAGCATCATAAGCAG-3’ |
| Elongation factor 1-alpha | Forward qPCR primer | 5’-TTGGAGGAATCTCCCAACATG-3’ |
|  | Reverse qPCR primer | 5’-AACGGACTTGACTTCAGTAGTC-3’ |
| Ubiquitin | Forward qPCR primer | 5’-AACGATAATGGGACCCGGTG-3’ |
|  | Reverse qPCR primer | 5’-GATTGGGATCAGTCAGCATTG-3’ |
| GAPDH | Forward qPCR primer | 5’-TCAACGGCAAGCTCACTGGT-3’ |
|  | Reverse qPCR primer | 5’-ACAAAATTCCCGTTCAAAGGAATC-3’ |
